# A Rare Cause of Esophageal Dysphagia – Secondary Esophageal Tuberculosis

**DOI:** 10.7759/cureus.21019

**Published:** 2022-01-07

**Authors:** Vidya Baleguli, Shahraiz Rizvi, Merin Varghese, Jawad Ilyas

**Affiliations:** 1 Internal Medicine, Northeast Georgia Medical Center, Gainesville, USA; 2 Infectious Disease, Northeast Georgia Medical Center, Gainesville, USA; 3 Gastroenterology and Hepatology, Northeast Georgia Medical Center, Gainesville, USA

**Keywords:** endoscopy, egd, mycobacterial infection, mycobacterium, odynophagia, dysphagia, tuberculosis, esophagus

## Abstract

Tuberculosis (TB) is an infectious disease caused by *Mycobacterium tuberculosis*. It continues to be one of the most common causes of death in adults across all countries. It is found to be relatively lower in North America. When aerosol droplets that contain *Mycobacterium* *tuberculosis* are inhaled, it can deposit in the respiratory tract, particularly in the patient’s lungs. Following this deposition, one of the four outcomes can take place. These include clearance of the organism immediately, primary disease, latent infection, and reactivation disease. Unhindered bacterial growth after primary infection can lead to a hematogenous spread of bacilli to produce disseminated TB. Esophageal involvement causing esophageal TB can be primary or secondary esophageal TB.

We present a unique case of secondary esophageal TB with symptoms of dysphagia and odynophagia with primary TB focus on the lung. Computed tomography (CT) of the chest noted diffuse bilateral miliary lung disease. TB QuantiFERON gold and sputum culture were positive for TB. Mycobacterial culture for identification with high-performance liquid chromatography showed isoniazid-resistant TB. The patient was started on antitubercular therapy with rifampin, ethambutol, moxifloxacin, and pyrazinamide for a total of nine months. Esophagogastroduodenoscopy (EGD) reported severe ulcerations of the oropharynx and focal ulceration in the proximal to the mid esophagus. Histopathology revealed active ulcerative and granulomatous esophagitis with mycobacterial organisms. After EGD she was started on a full liquid diet and advanced as tolerated. After discharge, she followed with the Health Department and had three negative sputum cultures after the completion of therapy.

## Introduction

In 2020, an estimated ten million people fell ill with tuberculosis (TB) worldwide. In the same year, a total of 1.5 million people succumbed to the same disease. Among the leading causes of death worldwide, TB is the 13th and the second leading infectious killer after COVID-19 [1] as per the World Health Organization (WHO).

When aerosol droplets that contain *Mycobacterium tuberculosis* are inhaled, they can deposit in the respiratory tract, particularly the patient’s lungs. Following this deposition in the lungs, we can have four different outcomes. These include clearance of the organism immediately, primary disease, latent infection, or disease reactivation. It is determined that at least one out of every 10 who are infected with *Mycobacterium tuberculosis* may develop an active infection at some point in their lifespan [2,3]. Unhindered bacterial growth following the primary tubercular infection can lead to a hematogenous spread of the bacilli to produce disseminated TB [4].

TB has been known to affect multiple organ systems. Gastrointestinal TB accounts for 1%-3% of all the cases globally. Potential areas of involvement are the esophageal, gastric, gastroduodenal, small and large intestinal, anorectal, and peritoneal [2,3]. We present a unique case of secondary esophageal TB with symptoms of dysphagia and odynophagia with primary TB focus in the lung.

## Case presentation

A 24-year-old female with a history of incarceration and polysubstance abuse presented with a one-month duration of dysphagia and odynophagia, worse with solids than liquids, associated with low-grade fevers, dry cough, night sweats, anorexia, sore throat, and a 40-pound weight loss. She had multiple outpatient visits in the month prior to her admission to the hospital. On review of these documents, it was noted that she had multiple complaints including sore throat, dysphagia, dental pain, and urinary tract infections (UTIs). She was treated with various antibiotics including amoxicillin suspension, Flagyl, and amoxicillin-clavulanate for possible dental abscess, Ludwig angina, and UTI with no significant improvement. Outpatient work-up included throat culture with no beta streptococcus, negative monospot. Computed tomography (CT) soft tissue neck without contrast showed markedly abnormal appearance of the lungs, a solid-appearing lesion within the right upper lung with multiple cavitary lesions seen within the lung apices. Differential considerations included septic emboli, miliary TB with cavitary lesions, atypical pneumonia, less likely pneumocystis pneumonia, and possible reactionary pneumonitis secondary to exogenous substances such as exposure to chemicals or drugs. The examination was limited secondary to the lack of IV contrast. No gross abnormality was seen within the visualized portions of the esophagus. She then underwent FL barium swallow, but the patient was unable to swallow the 13-mm-diameter barium pill.

**Figure 1:**
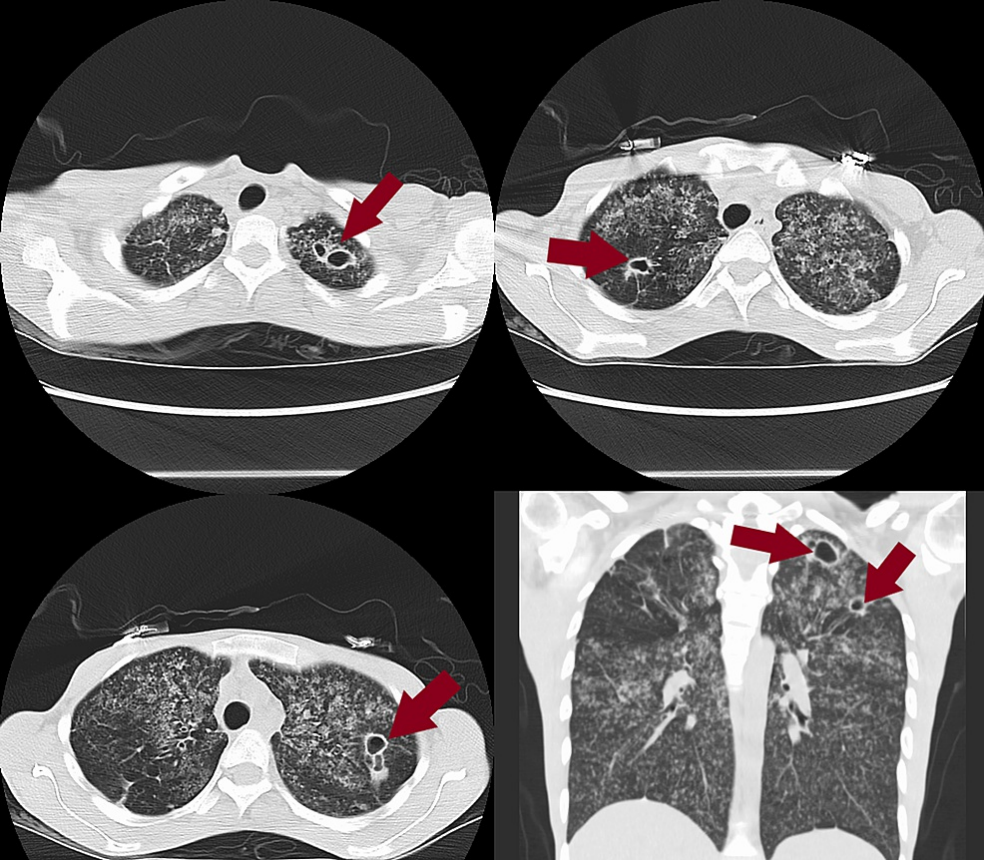
CT chest. Diffuse bilateral lung disease with areas of cavitation (red arrows). CT, computed tomography.

To further access the lesions in her lungs she had a CT chest with no contrast which showed extensive diffuse bilateral miliary lung disease with areas of cavitation (Figure 1). Miliary TB and fungal pneumonia were favored in the differential diagnosis. Miliary sarcoidosis, pneumoconiosis, Langerhans cell histiocytosis, and hypersensitivity pneumonitis were also considered. During her follow-up out-patient visit after all the imaging, she reported exposure to *Mycobacterium tuberculosis* a few years back. Interferon-gamma release was tested which returned positive. HIV1 + 2 AB + P24G was non-reactive.

**Figure 2:**
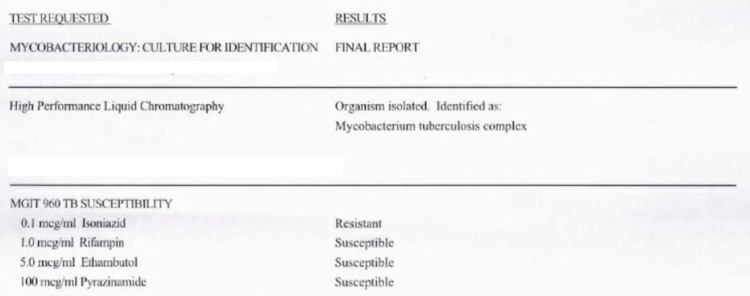
Mycobacterial culture for identification with high-performance liquid chromatography showed isoniazid-resistant tuberculosis.

As her dysphagia continued to worsen, she came to the hospital. Her vitals were significant for a blood pressure of 96/60 mmHg, temperature of 37.4°C (99.3°F), heart rate of 121 beats per minute, respiratory rate of 16 breaths per minute, oxygen saturation of 100%, and weight of 35.2 kg (77lb 9.6 oz), with BMI being low at 12.9. On examination, the patient was cachectic, pale with dry mucosa, white exudate on the oropharynx, but no swelling of the uvula or oropharynx, poor dentition, no fluctuance to the gumline, and fractured dentition to the left lower molars. Lung auscultation revealed normal breath sounds. The abdomen was soft, non-tender, and non-distended with normal bowel sounds. Laboratory work-up was significant for a white blood cell count of 10.2 k/uL (4.8-10.8 k/uL), hemoglobin of 9.7 g/dL (12.0-16.0 g/dL), and hematocrit of 32.7% (37.0%-47.0%). Inflammatory markers were elevated, with erythrocyte sedimentation rate at 127 mm (0-20 mm) and C-reactive protein at 11.20 mg/dL (0.00-0.60 mg/dL). Acid-fast bacilli (AFB) culture grew *Mycobacterium tuberculosis* and *Mycobacterium tuberculosis* polymerase chain reaction (MTB PCR) returned positive. Mycobacterial culture for identification with high-performance liquid chromatography showed isoniazid-resistant TB (Figure 2).

In-patient repeat CT of the chest was significant for diffuse bilateral miliary lung disease with areas of cavitation as previously seen (Figure 1). After an in-depth discussion with the state health department the patient was started on anti-tuberculous therapy with rifampin 600 mg PO once a day, ethambutol 600 mg PO once a day, pyrazinamide 800 mg PO once a day, moxifloxacin 400 mg PO once a day, and IV pantoprazole. Due to ongoing dysphagia, gastroenterology was consulted. The differentials included candidiasis versus severe esophagitis as the patient smoked Roxicodone for the past two years and there was questionable chemical burn/insult to the esophagus. She was started on aluminum hydroxide magnesium hydroxide simethicone, Benadryl, lidocaine, and sucralfate suspension. As there was no improvement over 48 hours with the interventions, esophagogastroduodenoscopy (EGD) was considered.

**Figure 3:**
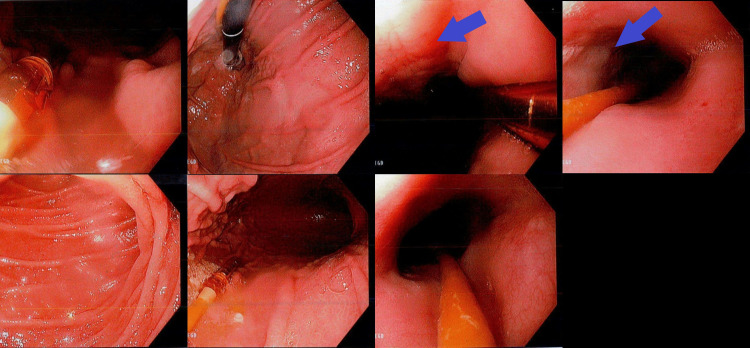
Esophagogastroduodenoscopy. Severe ulcerations of the oropharynx and focal ulceration in the proximal esophagus.

**Figure 4:**
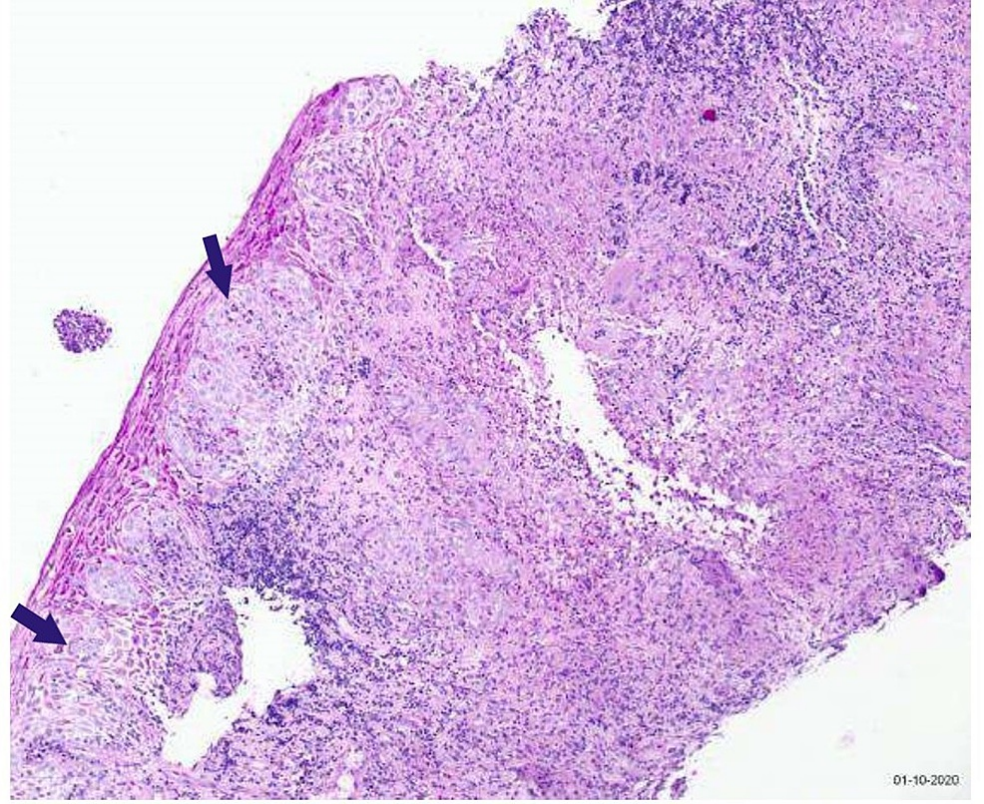
Tissue pathology. Esophageal biopsies showed active ulcerative and granulomatous esophagitis with mycobacterial organisms. The esophageal submucosa displayed multifocal, confluent, non-caseating, epithelioid histiocytic granulomas with focal Langerhans' type giant cells (blue arrows). The acid-fast bacilli and immunohistochemical mycobacterial stains displayed infrequent mycobacterial organisms.

EGD showed severe ulcerations of the oropharynx and focal ulceration in the proximal to the mid esophagus (Figure 3). Esophageal biopsies showed active ulcerative and granulomatous esophagitis with mycobacterial organisms. Furthermore, the esophageal submucosa displayed multifocal, confluent, non-caseating, epithelioid histiocytic granulomas with focal Langerhans' type giant cells. The AFB and immunohistochemical mycobacterial stains displayed infrequent mycobacterial organisms (Figure 4).

After EGD she was started on a full liquid diet and advanced as tolerated, continued on sucralfate and pantoprazole IV. In the following days, she was tolerating oral intake well and was considered stable for discharge. She was discharged home with pantoprazole, sucralfate, and anti-tubercular treatment with rifampin, ethambutol, pyrazinamide, and moxifloxacin for a total of nine months. After nine months of treatment, the patient had three sputum cultures that tested negative for AFB.

## Discussion

The only known reservoir for the bacterium *Mycobacterium tuberculosis* is the homo sapiens and the most common pathway for the transmission of this disease is from aerosolized droplets. It is a global health concern even though in developed countries it is rare to come across gastrointestinal TB. In developed countries, it has been reported in immunocompromised and immigrants from high-risk areas. Esophageal TB is a rare disorder to come across and accounts for 2.8% of all gastrointestinal TB [5]. Esophageal involvement by TB usually occurs at the level of the carina near the middle third of the esophagus. It can be further divided into two types, primary and secondary esophageal TB. Primary TB solely affects the esophagus. It is quite rare to come across primary TB. Secondary esophageal TB is correlated with a primary TB focus like the lungs or the mediastinal lymph nodes and can spread from them to the esophagus either via swallowed sputum, directly from the spine, mediastinal lymph node, tubercular lung cavity, retrograde spread from lymphatic drainage, or direct spread from the blood. Esophageal TB can often present as difficulty in swallowing and, if left untreated, it can cause deleterious complications like bleeding, perforation, fistula formation (esophago-pleural, esophago-bronchial, esophago-tracheal), aspiration pneumonia, hematemesis, traction diverticula, and esophageal strictures [6-8]. It can present with non-specific findings on the radiograph of the chest, but CT of the chest can show distinctive tuberculous lymphadenitis [6]. Diagnosis can be confirmed with a biopsy specimen histopathology and a positive MTB PCR [7].

At times, multiple esophageal tissue biopsies may be required to identify tuberculous granulomas as the density of tuberculous granulomas in the affected organ tissue may be low. Moreover, as tuberculous granulomas are in the submucosal layer, they may not be satisfactorily represented in the endoscopic mucosal tissue biopsies. In cases where there is presumed suspicion for esophageal or intestinal TB, it is crucial to obtain multiple deep tissue samples [8-10].

The United States Food and Drug Administration (FDA) has approximately 10 drugs that are currently approved for TB management. Among them, the first-line agents include isoniazid, rifampicin, pyrazinamide, and ethambutol, which are considered the mainstay of treatment. These four drugs are prescribed for two months, followed by a four to six months treatment duration with isoniazid and rifampin. Drug-resistant mycobacterial TB infection can be treated with fluoroquinolones, examples of which include levofloxacin, moxifloxacin, and gatifloxacin. These drugs can also be used for treatment if the patient is intolerant toward the first-line agents. Though these drugs are currently approved by the FDA for treatment of TB, it is commonly used to treat the same [11-14].

Single-drug resistance to isoniazid is by far the most common form of drug resistance in the United States. Among all the positive MTB cultures in 2019, approximately 9.6% of cases were found to be isoniazid resistant. There was a clear increase from the 8.4% of the cases in 2006 [15].

The general treatment recommendations for isoniazid-resistant TB are to use the first-line antitubercular agents. World Health Organization (WHO) specifically recommends rifampicin, ethambutol, and pyrazinamide for a total of nine months. They have also advised adding fluoroquinolone if the strain has simultaneous resistance to ethambutol or pyrazinamide [16]. The American Thoracic Society recommendations are like that of the WHO. They particularly recommend rifampicin, pyrazinamide, and ethambutol for nine to 12 months and at the same time have stated that a fluoroquinolone “may be added” [17]. In 2008, Menzies D, et al. [18] did a systematic review of retreatment and treatment of isoniazid resistance without multidrug resistance. They found no trials and only six cohorts in which WHO's recommended retreatment regimen was assessed, only nine trials focused on isoniazid resistance or retreatment cases, and no two trials made the same pair-wise comparison of regimens, precluding pooling [18].

The study by Medea Gegia et al. [19] in total identified 19 cohort studies and 33 randomized trials, which allowed them to pool results from 3744 patients with isoniazid-resistant TB who were being treated with a wide range of regimens. It allowed comparison of the outcomes among 19 ,012 patients with drug-susceptible TB treated at the same centers and with the same regimens. “On the basis of this review, isoniazid resistance is associated with increased treatment failure, relapse, and acquired multidrug resistance in patients treated with regimens containing only first-line tuberculosis drugs. Treatment with the standardized regimen recommended for new patients without drug-susceptibility testing could contribute substantially to the epidemic of multidrug-resistant tuberculosis, particularly in settings with a high prevalence of initial isoniazid resistance.” The outcomes of the study by Medea Gegia et al. suggest that rapid and accurate detection, and also, more applicable, effective treatment of isoniazid-resistant TB should take precedence [19].

Surgical interventions are indicated for the related complications from gastrointestinal TB including fistulas, strictures, and perforations that are resistant to medical and/or endoscopic therapy [6]. 

Our patient with secondary esophageal TB from a primary lung focus was treated with rifampin, ethambutol, pyrazinamide, and moxifloxacin instead of the standard first-line regimen as per the suggestions from The American Thoracic Society and the WHO [16,17]. Moxifloxacin was substituted for the standard isoniazid as the patient had an isoniazid-resistant *Mycobacterium tuberculosis* infection. Following nine months of treatment, she was followed up with sputum cultures that returned negative for AFB with improvement in her dysphagia.

## Conclusions

We present a unique case of secondary esophageal TB. It is important to keep esophageal TB in the differential in a patient with a history of exposure to *Mycobacterium tuberculosis* who presents with dysphagia and/or odynophagia. A prompt and accurate diagnosis can result in substantial therapeutic benefits, as observed in our patient, and decrease the associated mortality and morbidity if treatment is proper and promptly applied.
